# Evaluation of the Teaching Recovery Techniques intervention among newcomer students in Swedish schools: a randomised controlled trial turned into a feasibility study

**DOI:** 10.1186/s12889-024-19412-x

**Published:** 2024-07-17

**Authors:** Natalie Durbeej, Raziye Salari, Anna Sarkadi, Reeta Kankaanpä, Ilse Derluyn, An Verelst, Fatumo Osman

**Affiliations:** 1 Department of Public Health and Caring Sciences, Uppsala University, Uppsala, Sweden; 2 Faculty of Social Sciences/Psychology, Tampere University, Tampere, Finland; 3 Centre for the Social Study of Migration and Refugees, Department of Social Work and Social Pedagogy, Ghent University, Ghent, Belgium; 4 School of Health and Welfare, Dalarna University, Falun, Sweden

**Keywords:** Mental health problems, Post-traumatic stress disorder (PTSD), Newcomers, Teaching recovery techniques (TRT), School context

## Abstract

**Background:**

During recent years, Europe has faced the arrival of migrants whereof a considerable group of youth present mental health problems, such as symptoms of post-traumatic stress disorder (PTSD). Schools offer a safe environment for mental health interventions to these groups, yet there is limited research on the impact of school-based interventions addressing mental health problems in newcomer youths, especially in the Swedish context. This cluster randomized controlled trial (RCT) aimed to explore the effectiveness of the Teaching Recovery Techniques (TRT) intervention among newcomer students with PTSD symptoms in Swedish secondary schools.

**Methods:**

Nine schools were randomly assigned to TRT or a wait list control group prior to the baseline assessment. Follow-up data were collected immediately following the intervention and three months post-intervention. In total, 531 students were approached, of which 61 gave consent and were eligible to be included in the study: 55 in TRT and 6 in the control condition. Given the low number of participants in the control condition, we merely analyzed students who had received TRT.

**Results:**

We report on feasibility of recruitment, data collection, intervention delivery and intervention effectiveness. In terms of intervention effectiveness, within subjects ANOVAs revealed significant reductions in PTSD symptoms and general mental health problems from baseline to the three months-follow-up (*p* < 0.001).

**Conclusions:**

Our results indicate that TRT is a promising school-based intervention for newcomer students with PTSD symptoms. For a successful implementation of TRT in the school context, schools need to be engaged and the implementation should be managed by a local coordinator.

**Trial registration:**

ISRCTN, ISRCTN48178969, Retrospectively registered 20/12/2019.

## Introduction

During the past decades, migration has increased considerably, with many people residing in a country other than their country of birth. In 2020, 281 million people worldwide resided in a country other than their country of birth, and about 41 million of them (16.4%) were children [1]. Reasons for migration include challenging living conditions in the country of origin, such as conflict, violence or human rights violations, or economic and work-related motives. Although Sweden has a long-standing experience in receiving and accepting migrants, the country is still facing many challenges in delivering quality services to these populations [2].

Extensive scholarly evidence has indicated elevated rates of mental health problems from 10 to 50% in migrant children, compared to non-migrant peers, such as symptoms of posttraumatic stress disorder (PTSD), depression and anxiety [3–7]. These problems also pose a risk to developing mental health problems later in life [8]. Furthermore, research has shown that migrant girls report more anxiety and depressive symptoms than boys, whereas, in contrast, boys indicate more behavioral problems compared to girls [9, 10]. In some studies, older migrant children have also demonstrated higher levels of mental health problems compared to younger children [9].

Risk factors for developing mental health problems in migrant children are commonly divided into pre-migration/migration and post-migration factors. Pre-migration and migration factors generally include traumatic experiences, such as exposure to physical and sexual violence, threat, persecution and captivity, separation from family members, and experiencing the death of family members [11–16]. Post-migration factors include exposure to discrimination, social isolation and exclusion, material and financial stress, resettlement uncertainty, and inadequate or uncertain living conditions [15–18]. Altogether, migrant children are at risk of mental health problems. From a public health perspective, it is particularly important for host countries to quickly and efficiently address these problems through appropriate interventions targeting risk factors for mental health problems [14].

Several mental health interventions are available for refugee and migrant children, whereby Trauma-Focused Cognitive Behavioral Therapy (TF-CBT) is generally the recommended method for reducing PTSD symptoms and other mental health problems in these populations [19, 20]. In addition, schools have been identified as an ideal setting for delivering mental health interventions to these populations [21–23]. More specifically, schools provide an opportunity to easily reach children who may otherwise be isolated or unable to access services [24–27]. Further, schools are central to the children’s social network and a main contact point with the host society. Mental health interventions delivered in a school context may also promote positive intercultural relationships and facilitate the development of healthy behaviours [26–29]. Although the evidence base for the effectiveness of school-based interventions is rather limited, previous research has shown that such interventions are beneficial in reducing trauma-related symptoms among refugee and migrant children [24, 25, 30].

## Teaching Recovery Techniques

The Teaching Recovery Techniques (TRT) is a manualized group-based mental health intervention developed by the Children and War Foundation in the United Kingdom and Norway [31]. TRT is based on TF-CBT and was specifically designed to meet the needs of children in low-resource settings. The program includes both youth and caregiver sessions, aiming to promote coping and recovery from PTSD symptoms in children eight years or older and with traumatic experiences.

The TRT intervention has been used effectively in several contexts, such as Palestine [32], post-tsunami Thailand [33], and Iraq [34]. Reduced posttraumatic stress symptoms following TRT have been demonstrated among children and adolescents in these settings [32–34]. A study with unaccompanied refugee children in a community setting in Sweden also showed reductions in PTSD and depression symptoms after the TRT intervention [35]. Approximately 22% of the children recovered from PTSD symptoms, 63% remained unchanged, and few others slightly improved or deteriorated.

TRT has also previously been applied in school settings. More specifically, when TRT was applied in a school setting within the context of ongoing violence in Nablus, Palestine in 2010, large effect sizes were found for PTSD symptom reduction in children who participated in the program [36]. Another study in the United Kingdom with refugee and migrant children detected similar results, however, at the two-month follow-up, no long-term effects on PTSD symptoms were reported as only a few children completed the follow-up assessment [37]. According to our knowledge, TRT has not yet been evaluated in a Swedish school context.

Despite the potential for Sweden to reduce social inequities through its healthcare and social services systems, unmet needs widely remain with particularly mental health problems among migrant children [38]. As outlined above, schools have the potential to serve as a facilitator in delivering interventions such as TRT for reducing mental health problems in migrant children [25]. Nonetheless, there is limited research on the impact of school-based interventions targeting mental health problems in migrant youth, especially in the Swedish context. This study aimed to explore the effectiveness of the TRT intervention among newcomer students with PTSD symptoms in Swedish secondary schools.

## Methods

### Study design

The study is part of the EU Horizon 2020–funded RefugeesWellSchool (RWS) project, a collaboration between universities in six European countries that aims to evaluate various school-based interventions for promoting mental health among refugee and migrant children and youths [39]. The current study was planned as a cluster-randomized control design, with secondary schools randomized to either an intervention arm or a control arm in the form of a waiting list. However, due to restrictions imposed by the COVID-19 pandemic and constraints of resources, we encountered some implementation difficulties and were not able to run the project and consequently the analyses as planned (see below). Thus, we present the results in terms of the study’s feasibility. The study was approved by the Regional Ethical Review Board in Uppsala, Sweden (Dnr: 2019–031160) and registered with an international trial registry (ISRCTN 48,178,969). All participants of the current study signed a written informed consent form prior to participation. The informed consent form was available in 18 languages, which guaranteed that all participants were informed about the project and their participation rights and that they could sign their participation willingly in their native language. In accordance with Swedish legislation on informed consent procedures with minors, additional written informed consent from parents or guardians was also obtained for participants below 15 years of age. The study was conducted in a manner to allow for strict adherence to the CONSORT reporting guidelines.

### Planned sample size

The study aimed to recruit 20 Swedish schools with about 35 eligible students in each school resulting in 700 students in total [39]. Attrition rate was expected to be 20%.

### Setting and recruitment

The study was conducted in secondary schools in both urban and rural areas of Sweden. Schools that met the inclusion criterion of having a multi-ethnic profile, i.e., having at least 30% of its registered students with a non-Swedish background, were eligible to be included in the study. We used publicly available information on municipal websites to identify potentially eligible schools, then contacted the schools and informed them about the project.

Randomization of schools was conducted prior to the baseline assessment with an allocation ratio of 1:1 and through an online, third-party central randomization service named Sealed Envelope (www.sealedenvelope.com). The choice to randomize schools prior to the baseline assessment was based on a request made by the schools who wished for early information on which group they had been randomized to, in order to prepare for their participation in the project.

One person of the research team, blinded to the schools, ran the randomization process. This member had no previous information regarding the schools e.g. on various school-related challenges, nor any relations with school principals. Thus, the blinded randomization was done to prevent any subjective biases in relation to the project and the study outcomes.

Following randomization, schools were informed about the results and baseline data assessment was planned collaboratively. Students in grades seven to nine were given written and oral information about the RWS project and were invited to participate. Oral information about the project was provided to students during school hours by their teachers and/or members of the research team. Furthermore, students were informed that their answers would remain confidential and that they could withdraw from the study at any time, without further explanation.

Students were eligible to be included in the study if they (1) consented to participation in the study (legal guardians’ consents were required for youth younger than 15 years of age), (2) had been in Sweden not more than 6 years at the time of the study, (3) screened positive for PTSD symptoms (≥ 17 points) on the Children’s Revised Impact of Event Scale-8 (CRIES-8) [40] which was administered during the first data collection, and (4) were not receiving other mental health therapeutic interventions.

Parents of students participating in the study were also eligible to participate as the TRT intervention involves sessions for caregivers (see description under the Intervention section).

### Data collection procedure

Study outcomes were collected at three time points: baseline, prior to the intervention beginning (T1), the first follow-up, immediately following the intervention (T2), and the second follow-up, three months post-intervention (T3).

For students, data collection was conducted in classrooms at each school. A questionnaire that included the measures and variables used for the current study (see below) was developed. The questionnaire was completed independently on paper or online via computers or cell phones, using the secure platform LimeSurvey. Regular teachers and language teachers were present to help with the completion, if needed.

It was planned that parents/guardians who agreed to participate in the study would be contacted by mail/phone and would have the option to either fill in the survey online or receive a paper version through mail.

### Outcome measures

Full details of study outcomes have been published in the study protocol [39]. Primary outcomes for the students and parents as described in the study protocol were PTSD symptoms (Children’s Revised Impact of Event Scale; CRIES-8 [40]) and mental health problems (General Health Questionnaire; GHQ-12 [41]) respectively. The secondary outcomes for students included (1) measures of general mental health problems (Strengths and Difficulties Questionnaire, SDQ [42]), experience of the amount of stressors in daily life (Daily Stressors questionnaire; DSSYR [43]), positive development and resilience (Child and Youth Resilience Measure; CYRM-12 [44]), depression severity (Patient Health Questionnaire-9; PHQ-9 [45]), anxiety symptoms (Generalized Anxiety Disorder; GAD-7 [46]), and wellbeing (measured by one item developed for this study); (2) measures of social support and school belonging including social support (Multidimensional Scale of Perceived Social Support [47]), presence of interethnic friendships and friendship satisfaction (measured by a set of questions developed for RWS project), experience of discrimination (The Perceived Ethnic Discrimination Questionnaire [48]), and feelings of school belonging (Psychological Sense of School Membership Scale [49]), and (3) one measure of cognitive functioning i.e., perception of one’s own executive functions (Amsterdam Executive Function Inventory; AEFI [50]).

In the current study, we used the CRIES-8 total score [40] and the SDQ total difficulties score [42] as outcome measures. At baseline, the Cronbach alpha values for the CRIES-8 total score and SDQ total difficulties score were 0.86 and 0.73, respectively.

We also collected information about a number of variables that could be used as covariates to adjust for their potential confounding effects, as these variables have previously been associated with mental health problems in migrant populations [15, 51–53]. The covariates included factors related to circumstances before, during, and after migration: migration motive (fleeing war/parents came temporarily or permanently for work/to be reunited with parents or family/fleeing persecution or danger not because of war/I don’t know), migration status (permanent residence/temporary residence/decision pending/citizenship/undocumented/I don’t know), having been unaccompanied during the migration (yes/no), and having experienced family separation during the migration (yes/no). In addition, we adjusted the results for gender (boy/girl) and age (continuous variable). All covariates were assessed via separate items.

### Intervention

The Teaching Recovery Techniques (TRT) is a manual-based group intervention based on a TF-CBT (Trauma Focused – Cognitive Behaviour Therapy) approach to PTSD symptoms, however, no therapeutic experience is required to deliver the intervention [31]. TRT incorporates seven sessions for youth carried out throughout seven consecutive weeks. Each session lasts between 90 and 120 min and includes skills training and homework.

In the first session for adolescents, participants familiarise with each other and try to visualize a safe space. The focus of the second and third sessions is on the intrusion spectrum of PTSD. During these sessions, the participants continue to visualize a safe space. In addition, they are asked to think of or draw their experiences of war-related events and common trauma reactions, and to share them with each other and the group leaders. The purpose of these activities is to normalize the youth’s experiences and reactions.

The fourth session centres on the arousal symptoms and techniques for relaxation, whilst also exploring possible coping mechanisms. During the fifth session, the group plans and practices graded exposure in real-life. Throughout the sixth session, the adolescents have the opportunity to expose themselves to their trauma by utilizing previously learnt coping strategies and writing, drawing or talking to others. In the last session, which serves as a follow-up, space is given for conversations without further deepening the content learnt from sessions one to six.

Additionally, two sessions for the participating adolescents’ caregivers are provided, the first prior to the beginning of the youth sessions and the second between the second and fourth youth sessions. The rationale behind the caregiver sessions is to provide information on the TRT content and to guide parents in acquiring self-help strategies to extend the supportive environment of their child beyond the school context to the home.

In the current study, a fidelity checklist on paper was distributed to all group leaders in order to promote and monitor adherence to the core design of the intervention.

### Statistical analyses

Given the low number of participants in the control condition (see below), original planned analyses were deemed inappropriate. Thus, we opted to focus on the study’s feasibility.

All analyses were conducted using IBM SPSS, version 27. Descriptive statistics were used to describe the participants, presented with frequencies, proportions, means, and standard deviations. The intervention effectiveness was assessed by examining within-subject changes from baseline, to post-intervention and three months follow-up using within subjects ANOVA-tests along with Tukey’s post hoc tests. P-values < 0.05 were considered as statistically significant.

Prior to the analyses, multiple imputation was used to impute the missing data at the post-intervention and three-months follow-up assessments. Multiple imputation is considered a good strategy for dealing with missing data and has three phases: (1) Imputing the data in *m* datasets; (2) Analysing the data in each data set; and (3) Pooling the results of all data sets to come to inference. In the current study, data were imputed in five datasets and we report the pooled results, however, since SPSS does not provide the F-statistics on the pooled data, we used the combination rules as described by van Ginkel and Kroonenberg [54], using a SPSS macro to calculate the F-statistics based on the pooled data.

Furthermore, we used multiple linear regression models, controlling for baseline scores, to test associations between factors related to circumstances before, during, and after migration and outcomes. SPSS provides regression coefficients and confidence intervals based on pooled data, which are presented in the Results section. Prior to computing the regression models, the data were checked for multicollinearity by examining the variance inflation factor (VIF) values for all covariates [55]. VIF values ≥ 10 indicate multicollinearity. The VIF values for the covariates in the current study ranged from 1.12 to 4.49, indicating that multicollinearity was not present in the data.

Finally, to assess the clinical significance of change in symptoms of PTSD and general mental health problems following TRT, we classified the participants as recovered, improved, unchanged or deteriorated based on the Reliable Change Index (RCI) and Clinically Significant Change (CSC) approach [56, 57]. This approach includes measures on whether the change in scores is larger than expected due to measurement error (statistical significance) and the participant’s shift from a clinical state to a non-clinical state (clinical significance).

## Results

### Feasibility of recruitment

Recruitments of schools started in August 2018 and occurred in intervals with about 10 schools being contacted in each time interval. Due to constraints imposed by the COVID-19 pandemic, the recruitment of school was first paused and then prematurely terminated in December 2020. In total, we contacted 72 schools who received information about the project and randomization procedure [39, 58]. Of 21 schools that showed interest in the study, three schools did not meet the eligibility criteria, five had other ongoing activities and four had ongoing organizational changes. Additionally, several schools that declined participation mentioned that schools should focus on students’ academic education, not on healthcare. In total, nine schools participated in the study and were randomized to the intervention (*n* = 5) or the control group (*n* = 4).

Two senior members of the research team had extensive contact with school principals building a working relationship and keeping them informed about the implication of the project for students’ school performance. Principals were prompted to communicate the information with the other school staff. However, we noted that the extent of information shared with staff varied greatly among participating schools. In some schools, staff were fully informed and highly engaged in informing their students about the project and encouraging them to participate, while others knew little about the project and had spent little time on preparing students for the information meeting. In general, the control schools showed less interest in recruiting participants, than the intervention schools.

The members of the research team visited schools to provide students with study information. In six out of nine schools, information sessions were held in students’ classrooms. In one school, students in grades seven and eight received the information in their respective classrooms, while students in grade nine were gathered in the school hall. In two schools, teachers handpicked students who met the inclusion criteria of having arrived to Sweden six years ago or less and gathered them in the school hall. Members of the research team started the session with describing the study and then informed students about what participation entailed. They then directed students to a table where study information was available in Swedish and seventeen other languages. About one month after information sessions, members of the research team visited the schools for the first data collection. Data collection was conducted in classrooms at each school and was completed between September 2019 and June 2020.

**Fig. 1:**
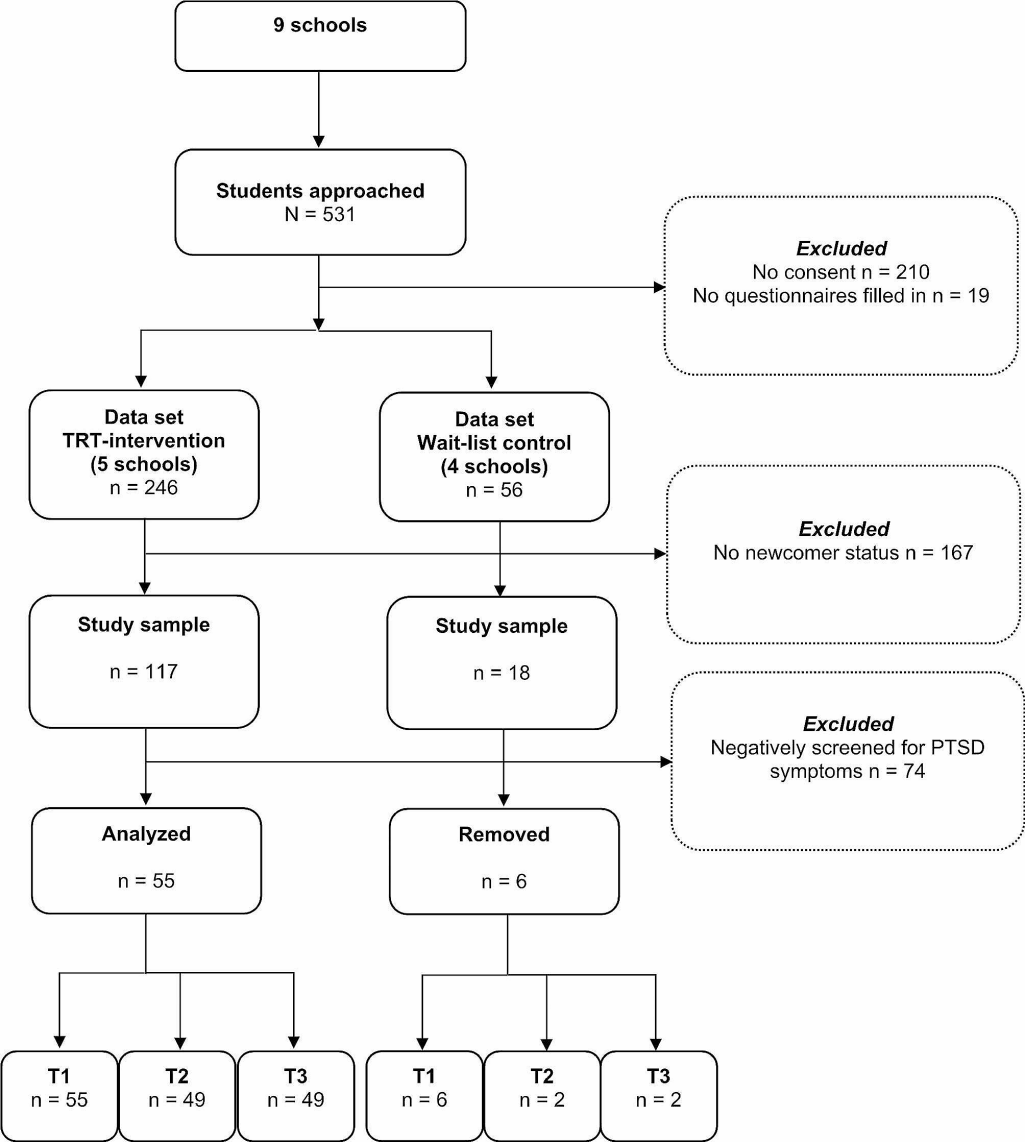
Participant flowchart

In total, students from 29 classes were invited to participate in the study. As described in the participant flow chart (Fig. 1), 531 students were approached for the study, of which 210 guardians and/or students did not consent to trial participation. Furthermore, for 19 students, no questionnaires had been filled in. Out of the 302 remaining students with valid consent forms, those with a newcomer status, defined as having been residing in Sweden less than or equal to 6 years, were selected. This led to a further exclusion of 167 study participants and thus a study sample of 135 students. Only those positively screened for PTSD symptoms with a CRIES-8 score of 17 or more were eligible to participate in the current study. This resulted in the inclusion of 55 participants in the intervention group and 6 participants in the control group. However, since the control group only included 6 students they were removed from the analysis. Thus, the sample of this study comprised 55 newcomer students from the intervention group who had received TRT. Among these, 49 students who had participated in both follow-up assessments were included in the main analyses.

### Feasibility of data collection

Completing each assessment took about 45 to 60 min. Observation reports from the assessment meetings indicated that most participants had difficulties in completing the assessments independently and needed help in understanding the questions, particularly items of the SDQ and definition of trauma in the CRIES-8. The questionnaires were available in 22 languages. No interpreter was booked for the assessment sessions, however, many of the members of the research team and schools teachers were able to speak languages other than Swedish and were able to help students if necessary. The questionnaires were completed in Arabic, Farsi, Tigrinya, Somali, French, Polish, English, and Swedish. We had planned to collect PHQ-9 and GAD-7 during the first TRT group, but this proved impossible due to limited time and resources available.

### Feasibility of intervention delivery

All group leaders of the TRT intervention, i.e., school teachers, school counsellors and school nurses, obtained a three-day training from the Swedish non-governmental organization Child’s Rights in Society (BRIS – Barnens Rätt i Samhället). However, only two of the five intervention schools started delivering TRT. These two schools had started running the TRT group before the pandemic unfolded and continued delivering the intervention even during the pandemic. One school delivered three TRT groups, while the other one delivered only one. The TRT sessions took place during the normal school days by either being embedded in the schedule or being classified as an optional course after ordinary classes. Each TRT group comprised 12 to 16 students. One group comprised students from the same class, whereas the other three comprised students of different ages and from various classes.

None of the group leaders returned the fidelity checklist to the research team after the program was finished. Thus, it was not possible to assess adherence to the program. The commencement of TRT groups in the other three intervention schools were interrupted by the pandemic. The teachers in these three schools never ran the TRT intervention in the group format, but reported using the knowledge learned to support students in other ways.

Although all parents in the intervention schools were invited to the TRT information meetings, the number of parents who participated in the meeting was relatively low. Parents of those youth who participated in TRT were invited to participate in the TRT sessions for caregivers, however no one took this opportunity. Some older adolescents reported they had not shared their difficulties with their parents to protect them. The reasons for parents’ non-participation were not explored further.

All group leaders were instructed to document the number of TRT sessions each participant attended, however, no one provided this information to the research team. Thus, we were also not able to assess attendance rates of the TRT program.

Narrative reports from teachers trained to deliver TRT showed that they were motivated because of the health needs they perceived; some of their students were negatively affected by what they had been through, and faced difficulties focusing on their studies [59]. The teachers expressed that receiving the TRT training provided them with tools that could support their interaction with the students who have experienced trauma. In addition, some emphasized the importance of informing other teachers about the ongoing TRT sessions. Because not all teachers knew about the ongoing TRT sessions, some students were not allowed to leave their classes. Therefore, the teachers who delivered TRT suggested that TRT should be scheduled as other classes and not as extracurricular activities. Another suggestion teachers had was to use a venue where students could relax and not feel like they were in a classroom.

Three of four TRT groups were delivered in Swedish; one of the teachers who delivered TRT could speak the students’ native language. Three interpreters were used in one of the groups, which made the sessions longer. However, the teachers who delivered TRT in this group reported that this had not affected the sessions or the group dynamic negatively.

### Intervention effectiveness

As mentioned before, given the low number of participants in the control condition, original planned analyses were deemed inappropriate. Thus, we limit the analyses to the CRIES-8 and the SDQ outcomes and the students in the intervention group only. We start by describing characteristics of 55 students with the baseline data available.

**Table 1:** Participants’ characteristics (*n* = 55)

Variables	*n* (%)
**Age**,** M (SD)**	15.5 (3.0)
**Gender**	
Boy	29 (52.7)
Girl	25 (45.5)
Missing data	1 (1.8)
**Country of origin**	
African countries^1^	24 (43.6)
Middle-east countries^2^	17 (30.9)
Other^3^	4 (7.3)
Missing data	10 (18.2)
**Migration motive**	
Fleeing war	28 (50.9)
Parents came temporarily or permanently for work	7 (12.7)
To be reunited with parents or family	6 (10.9)
Fleeing persecution or danger not because of war	10 (18.2)
I don’t know	4 (7.3)
**Migration status**	
Permanent residence	33 (60.0)
Temporary residence	3 (5.5)
Decision pending	3 (5.5)
Citizenship	7 (12.7)
Undocumented	2 (3.6)
I don’t know	5 (9.1)
Missing data	2 (3.6)
**Unaccompanied during migration**	
Yes	4 (7.3)
No^4^	49 (89.1)
Missing data	2 (3.6)
**Separated from family members during migration**	
Yes	17 (30.9)
No	37 (67.3)
Missing data	1 (1.8)
**CRIES-8 total score at baseline**,** M (SD)**	23.9 (5.1)
**SDQ total difficulties score at baseline**,** M (SD)**	14.6 (5.5)

^1^ Eritrea, Somalia, Ethiopia, Nigeria, Gambia, & Djibouti

^2^ Afghanistan, Syria, Saudi Arabia, Iraq, & Iran

^3^ Thailand & Kosovo

^4^ Accompanied with parents and siblings/only siblings/other relatives

The mean age of the sample was 15.5 years, and just over half of the participants were boys (53%) (Table 1). The majority of participants were born in African countries, and the most common migration motive was fleeing war (51%) followed by fleeing persecution or danger not because of war (18%). More than half of the sample had a residence permit (60%) and about a third (31%) had been separated from family members during the migration. However, the great majority of the students arrived in the host country accompanied by parents, siblings or other relatives (89%). The average CRIES-8 total score was 23.9 and the average SDQ total difficulties score was 14.6.

The within subjects ANOVA-tests revealed a reduction in both PTSD symptoms according to the CRIES-8 total score and general mental health problems according to the SDQ total difficulties score from baseline to three months-follow-up. There were no difference in CRIES-8 and SDQ scores between post-intervention and three months follow-up assessment (Table 2). The RCI and CSC analyses showed that 35% of the participants were classified as recovered on PTSD symptoms and 20% on general mental health problems at three months follow-up (Table 3). A few students deteriorated, whereas the majority were unchanged (65 and 63% for PTSD symptoms and general mental health problems, respectively) according to these measures. When exploring associations between factors related to circumstances before, during, and after migration and outcomes through multiple linear regression models, none were associated with the outcomes at the three months follow-up (Table 4).

**Table 2:** Differences in CRIES-8 total scores and SDQ total difficulties scores at baseline, post intervention and three months follow-up (*n* = 49)

Variables	Baseline mean (SD)	Post-intervention mean (SD)	Three months follow-up mean (SD)	Within subjects ANOVA-test	*p*-value^1^	Bonferroni correction for pairwise comparisons
**CRIES-8 total score**	23.9 (5.1)	11.7 (6.0)	11.9 (6.1)	F (2,96) = 97.3	< 0.001	B^2^ > P^3^, T^4^
**SDQ total difficulties score**	14.6 (5.5)	12.2 (6.2)	10.4 (5.4)	F (2,96) = 10.1	< 0.001	B > P, T

^1^ P-values indicating significant differences across all three measurement points

^2^ B = Baseline mean

^3^ P = Post-intervention mean

^4^ T = Three months follow-up mean

**Table 3:** Number of participants in each category at three months follow-up using the Reliable Change Index and clinical significance change approach (*n* = 49)

Category	PTSD symptoms (CRIES-8) *n* (%)	Overall mental health problems (SDQ) *n* (%)
Recovered	17 (34.7)	10 (20.4)
Improved	0 (0)	7 (14.3)
Unchanged	32 (65.3)	31 (63.2)
Deteriorated	0 (0)	2 (4.1)

**Table 4:** Multiple linear regression models for exploration of factors related to circumstances before, during, and after migration in relation to outcomes at three months follow-up (*n* = 49)

	Outomes			
	PTSD symptoms at three months follow-up (CRIES-8 total score)		General mental health problems at three months follow-up (SDQ total difficulties score)	
Independent variables	B (95%CI)	*p*-value	B (95%CI)	*p*-value
**PTSD symptoms at baseline (CRIES-8 total score)**	0.36 (-0.23-0.95)	0.218	-	-
**General mental health problems at baseline (SDQ total difficulties score)**	-	-	0.14 (-0.22-0.50)	0.449
**Gender**				
Boy (ref)				
Girl	0.69 (-3.96-5.33)	0.766	0.06 (-3,34 − 3,45)	0.973
**Age**	-0.09 (-0.78-0.61)	0.801	0.09 (-0.59-0.78)	0.779
**Migration motive**				
I don’t know (ref)				
Fleeing war	-0.04 (-7.93-7.85)	0.991	-1.93 (-8.90- 5.03)	0.586
Parents came temporarily or permanently for work	0.21 (-10.71-11.13)	0.970	1.02 (-6.89- 8.92)	0.801
To be reunited with parents or family	2.07 (-7.45-11.59)	0.669	-1.95 (-10.16-6.26)	0.641
Fleeing persecution or danger not because of war	-2.11 (-10.92-6.71)	0.639	-5.58 (-13.18-2.01)	0.150
**Migration status**				
I don’t know/ Temporary residence/Decision pending/undocumented (ref)				
Citizenship or permanent residence	1.14 (-4.35-6.64)	0.674	-2.22 (-6.09-1.65)	0.260
**Unaccompanied during migration**				
No (ref)^3^				
Yes	3.37 (-6.91-13.66)	0.505	3.38 (-6.28- 13.04)	0.260
**Separated from family members during migration**				
No (ref)				
Yes	2.09 (-2.91-7.09)	0.406	-2.14 (-5.85-1.58)	0.473

## Discussion

The current study aimed to evaluate the effectiveness of TRT in decreasing symptoms of PTSD and general mental health problems in newcomer students in Sweden. We planned the study as a cluster-randomized control trial, aiming to recruit 20 schools in Sweden. However, we found it difficult to recruit schools as only a few (*n* = 21, 29% of those contacted) showed interest in participating in the study and fewer met the eligibility criteria (*n* = 9, 13% of those contacted). Reasons noted for this difficulty were ongoing development projects, other ongoing activities or ongoing organizational changes. Previous studies have highlighted several factors hindering the implementation of targeted mental health interventions within schools [60, 61], including whether the intervention aligns with the school’s policies, goals, and philosophy. In our study, several schools that declined participation mentioned that schools should focus on students’ academic education, not on healthcare. A study within the RWS project has previously revealed that teachers do not view schools as an arena for treating students’ mental health problems [58].

However, as Gee and colleagues [60] mentioned, alignment of health and education in school policies is of immense importance and the health and education sectors should collaborate to promote students’ mental health. Moreover, the Swedish school policy does, in fact stipulate that schools should promote students’ mental health through school health services. Our observations, however, suggested that the school administrators, teachers and school health services had different views. We recommend that when mental health interventions are implemented in schools, principals and staff need to be engaged before the implementation is initiated. This will ensure that the intervention is tailored to the unique needs of the schools and the population of students in the schools [60].

Randomization may have been another reason for the low interest in the overall participation among schools, as well as the discrepancies in sample sizes between the intervention (*n* = 55) and control groups (*n* = 6). In general, the control schools showed little interest in recruiting participants, whereas the intervention schools showed more interest and engagement in participant recruitment throughout the project. Being randomized to a waitlist control condition implies receiving an intervention after the evaluation has been completed. Research suggests that schools in a control condition might require other incentives to participate and collect data [62]. For instance, recent studies have highlighted the importance of avoiding passive controls, noting that schools allocated as controls should be offered comparable interventions or other incentives to maintain a positive relationship [62–64]. Offering a comparable intervention or providing other incentives to the control schools (e.g., vouchers to buy school supplies) could potentially have led to a higher response rate among schools overall as well more participants in the control group.

Overall, the findings showed that teachers who delivered TRT sessions were very positive about implementing mental health interventions in school settings. However, they highlighted that (1) the TRT intervention should be integrated into the school curriculum, with sessions scheduled during school hours, and that (2) other school staff should be involved in the implementation of the intervention as well. For instance, the teachers perceived that their colleagues, who were not delivering the intervention, had little information about the project. Previous studies have also highlighted that a lack of support from teachers and school management can decrease the success of school mental health interventions [60, 65]. In our study, while most school principals were well engaged, we were not sure of the extent to which school principals engaged teachers in implementing the project or whether they provided any project information to teachers. This highlights the importance of a local coordinator in each school who could manage the program implementation, engage staff and deliver sufficient information to all parties involved.

Further, the school teachers emphasized the importance of having session venues that differed from classrooms to create a calm and safe environment for the students. Only four TRT groups were held in two schools. One reason was the interruption of COVID-19, which caused schools to not prioritize the TRT sessions. Although the TRT program includes two sessions for students’ caregivers, no caregiver sessions were held as the caregivers did not show interest or did not participate in TRT information sessions held for caregivers and students; this is similar to previous research conducted in Sweden [35].

In terms of intervention effectiveness, our results showed that both the PTSD symptoms and general mental health problems reduced significantly from baseline to the three-month follow-up. In addition, 35% of the participants were classified as having recovered from PTSD symptoms and 20% from general mental health problems, similar to what we found in the original pilot test of TRT in a community setting [35]. When exploring associations between factors related to the participants’ circumstances before, during, and after migration and the intervention outcomes, we found that none of the factors were associated with the outcomes at the three-month follow-up, which could be a result of the small sample size. Nonetheless, our overall findings are in line with our previous research investigating the effects of TRT [35] and suggest that TRT is a promising school-based intervention for newcomer students with PTSD symptoms who have resided less than six years in Sweden.

### Strengths and limitations

An obvious limitation of this study is its lack of a control group. Originally, the study used a cluster-randomized control design that included one intervention group and one control group. However, due to low participant rates and high dropout rates, the control group could not be included. Other methodological limitations relate to measurement issues. All data were based on self-reports. During the data collection, we noted that students had difficulties in responding to certain questions, and many needed assistance when completing the questionnaires. More specifically, students struggled to understand the SDQ measure, and respondents might have over- or underreported symptoms measured by SDQ’s items. This is in line with previous research suggesting that the SDQ should be used cautiously when assessing mental health problems among migrant populations as items might be over- or understated or difficult to understand [66]. Furthermore, the students had difficulties understanding to which traumatic events the CRIES items referred. Many of the students had experienced multiple traumatic events so the questionnaire’s instructions need to be adjusted to accommodate for this circumstance.

Another obvious limitation is that it was not possible to assess intervention adherence or attendance rates. Due to the low number of participants, we also could not adjust the results for school-related variables, such as school and class type, in the regression model. However, although none of the variables were associated with the outcomes at the three-month follow-up, we included various factors related to the participants’ circumstances before, during, and after migration that are relevant to the development of mental health problems in newcomer children. This should be regarded as a strength. Another strength is that the study included two post-intervention assessment points —namely, one immediately after the TRT intervention and one three months later. Lastly, the study was conducted in a school setting using regular school staff as TRT group leaders; as far as we are aware, no previous study has evaluated the TRT program in a Swedish school setting. Thus, our findings contribute to knowledge on this topic.

## Conclusions

Overall, our results indicate that TRT is a promising school-based intervention for newcomer students with PTSD symptoms in Sweden. Thus, schools could be regarded as a promising setting for delivering mental health interventions, such as the TRT program, for newcomer students in Sweden. However, prior to implementation of mental health interventions such as TRT, schools need to be engaged to ensure that the program is tailored to their contextual needs. It is also crucial to have a local coordinator in each school to manage the implementation, deliver information to staff and engage them, as school principals might not have the time to do these tasks. Further, when evaluating the effects of TRT in a trial, control schools need to be offered other interventions or incentives to enable the collection of data over time. Following these recommendations could lead to a successful implementation of TRT in a school setting and subsequently an improved opportunity to explore the effects of the program in randomized controlled trials.

## Data Availability

All relevant data are presented in the article. The study’s data cannot be publicly shared due to ethical restrictions, i.e. the data contain potentially identifying and sensitive information. This was imposed by the Regional Ethical Review Board in Uppsala. All relevant data are available upon reasonable request and approval from the Senior Registrar Clerk at Uppsala University. Interested researchers may contact the Principal Investigator, Associate Professor Natalie Durbeej (Natalie.Durbeej@uu.se) or Uppsala University (registrator@uu.se), to request the data.
